# Domestic violence and associated factors during COVID-19 epidemic: an online population-based study in Iran

**DOI:** 10.1186/s12889-022-12536-y

**Published:** 2022-04-15

**Authors:** Kamran Bagheri Lankarani, Camellia Hemyari, Behnam Honarvar, Elahe Khaksar, Fatemeh Shaygani, Mohammad Reza Rahmanian Haghighi, Mohammad Reza Shaygani

**Affiliations:** 1 Health Policy Research Center, Institute of Health, Shiraz University of Medical Sciences, Shiraz, Iran; 2 Research Center for Psychiatry and Behavioral Sciences, Shiraz University of Medical Sciences, Shiraz, Iran; 3 School of Mathematics and Statistics, Carleton University, 1125 Colonel By Drive, Ottawa, ON Canada; 4 Student Research Committee, Shiraz University of Medical Sciences, Shiraz, Iran

**Keywords:** COVID-19, Coronavirus, Domestic violence, Violence, Lockdown

## Abstract

**Background:**

The novel coronavirus disease 2019 has severely affected communities around the world. Fear and stress of being infected, along with pressure caused by lockdown, prevention protocols, and the economic downturn, increased tension among people, which consequently led to the rise of domestic violence (DV). Therefore, this study was conducted to determine the rate of change in DV and its associated factors during the COVID-19 epidemic in Shiraz, Iran.

**Methods:**

In this cross-sectional study, 653 individuals with the age of over 15 years from Shiraz were participated through snowball sampling and filled out an online questionnaire through the WhatsApp platform. A 51-item, self-administered and multidimensional (knowledge, attitude, and practice) questionnaire was designed and assessed 653 participants. The gathered data was analyzed using SPSS software (version 25), and variables with a *p*-value of less than 0.05 were considered statistically significant.

**Results:**

In this study, 64.2% of the respondents were within the age range of 31–50 years, and 72.6% of the subjects were female. Furthermore, 73.8 and 73.0% of the individuals were married and educated for over 12 years, respectively. The DV increased by 37.5% during the quarantine period, compared to before the pandemic. The emotional type was the most common type of violence; the sexual type was the least frequent. Multivariate analysis indicated that infection with COVID-19, drug use, high level of co-living observation of anti-COVID prevention protocols, and lower level of physical activity during the quarantine period had a positive and significant association with the occurrence of DV.

**Conclusion:**

Based on the obtained results, it is required to implement effective harm-reduction policies and measures in the community due to the increasing rate of DV during the COVID-19 epidemic.

## Background

Domestic Violence (DV) is a broad term referring to violent behaviors within families which may be physical, sexual, psychological, or financial [1]. Individuals who have undergone DV are at higher risk of developing physical and mental health problems and sexually transmitted infections [2]. Due to the global statistics, DV is generally experienced by 35% of all women, and there has been limited information about male victims [3]. In Iran, the prevalence rates of this phenomenon were reported as 53.7 and 40.4% among females and males, respectively [4]. Also, previous researches showed alcohol consumption, religion, growing up with DV, being young and childhood abuse increased risk of DV. On the other hand, higher education, formal marriage and high socio-economic status offered protection against DV [5, 6].

In the past, researchers have reported a link between natural disasters and an increase in DV which is supposed to be caused by stress due to factors such as physical imprisonment, financial problems and unemployment [7]. Currently, coronavirus disease 2019 (COVID-19), as one of the main public health challenges, has raised concern among individuals and communities worldwide [8]. In response to the coronavirus pandemic, the Iranian government ordered quarantine and lockdown of major cities including Shiraz to restrict coronavirus transmission. The reason for this was to slow down the spread of the infection and give the health system ample opportunity to pool resources to fight the infection. One of the unintended costs of this quarantine, as some studies have pointed out, is its impact on people’s mental health, including the potential for increased DV. According to researches on the prevalence of DV in Iran, 30 to 90% of women are abused or mistreated [4, 9–11]. In a meta-analysis conducted by Hajnasiri et al. with a sample size of 15,514 people, they determined that around 66% of Iranians were victims of DV using the random effects model (CI 95%: 55–77) [11]. 70% (CI 95%: 57–84) of DV was reported in the east of the country, 70% (CI 95%: 32–100) in the south (CI 95%: 56–94), 75% in the west (CI 95%: 56–94), and 62% in the north (CI 95%: 37–86). In another study, researchers in Bandar Abbas (a city located in the south of Iran) studied five hundred women and reported that 92% of them were subject to DV [12]. The author of another study found that 36% of DV is committed against married women, with 30% being physical and 29% psychological [13]. Since the outbreak of COVID-19, there has been a growing trend in DV rates in several countries, including China, the United Kingdom, Spain, Brazil, France, and the United States [13–15]. Although the surge in DV is temporary in line with the waves of coronavirus infection, its psychological effects are supposed to be long-lasting [16, 17]. On the other hand, due to the decrease in the number of people visiting governmental support centers and the difficulty of calling the emergency services in the presence of others, in some cases, the number of reports of domestic violence may inaccurately show a decreasing trend [18]. Without sufficient surveillance, it is impossible to precisely estimate the burden of DM. Indeed, due to the selection biases and lack of information, the rates of DV have been underestimated. This can be like an iceberg that will have many negative consequences for society in the future [19].

To the best of our knowledge, no study on DV during the COVID-19 outbreak in Iran has been conducted; thus, the current study aimed to determine the rate of change in DV after the COVID-19 pandemic compared to before it, and its associated factors in Shiraz, Iran. The purpose of this study was to investigate the knowledge and attitudes of participants about domestic violence, to determine the incidence of domestic violence during the Covid-19 lockdown of Shiraz, and to identify the factors associated with the increase in domestic violence in the quarantine period. To our knowledge, this study is the first to evaluate the impact of lockdown on the rate of DV during the COVID-19 pandemic in Iran.

## Methods

### Setting and participants

This cross-sectional research was conducted between April and July of 2020, one month after the Iranian government declared a lockdown in Shiraz, Iran. Shiraz, with a population of about 2 million people, is the capital city of Fars province in southern Iran. Although the sample size was calculated at 400 based on the anticipated DV prevalence of 50%, confidence interval of 95%, and error of 5%, it was increased to 600 based on snowball convenience sampling and an effect size of 1.5. Individuals having an average age of above 15 years and a Shiraz residence were eligible to participate in this study. Except for a lack of willingness to participate in this study, there were no exclusion criteria. The data collecting technique in this study was an online self-administered questionnaire, and the link to it was distributed to 20 people from various educational and social backgrounds using WhatsApp, Iran’s most popular social network. Each of them was asked to select five people to whom the questionnaire should be sent. The remaining individuals were contacted in the same manner until data saturation was achieved. The survey was completely anonymous.

### Data collection

The questionnaire contained 51 items divided into four sections: demographic information, knowledge, attitude, and practice. The first section included 25 questions about the participants’ demographic characteristics, as shown in Table 1. The second section comprised 12 questions about the public’s understanding of the term and different types of DV. The third section included two items about the respondents’ attitudes. In the second and third sections of the questionnaire, each item was rated on a 2-point Likert scale, with a total score of 12 being the sum of all the items’ values. Higher levels of knowledge and violence were indicated by higher scores in the second and third sections, respectively.

**Table 1:** Socioeconomic, demographic, anthropometric and medical backgrounds of participants (*n* = 653)

Variable	n (%)
**Age**	
15–30	189(28.9%)
31–50	419(64.2%)
> 50	45 (6.9%)
**Gender**	
Female	474(72.6%)
Male	179(27.4%)
**Marital Status**	
Single	171(26.2%)
Married	482(73.8%)
**Number of co-livings you have?**	
Mean ± Std	3.31 ±1.39
Median(min-max)	3(1–6)
**Level of Education(years)**	
≤12	176(27.0%)
>12	477(73.0%)
**Changing the state of physical activity?**	
Higher	88(13.5%)
No change	200(30.6%)
Lower	365(55.9%)
**Using tobacco (cigarettes, hookah, pipe)**	
Yes	92(14.1%)
No	561(85.1%)
**Using alcohol as a beverage**	
Yes	59(9%)
No	594(91%)
**Using drugs**	
Yes	10(1.5%)
No	643(98.5%)
**History of mental illness or medication (over the past year)**	
Yes	64(9.8%)
No	589(90.2%)
**History of physical illness (over the past year)**	
Yes	158(24.2%)
No	495(75.8%)
**Face-to-face communication with first-degree relatives who were living in other houses**	
> once a week	145(22.2%)
Once a week	110(16.8%)
Once in 2 weeks	108(16.5%)
< Once in 2 weeks	290(44.4%)
**Remote communication with first-degree relatives who were living in other houses**	
> once a week	500(76.6%)
once a week	89(13.6%)
Once in 2 weeks	25(3.8%)
< Once in 2 weeks	39(6%)
**How many times have you left home?**	
> = 3 times in a week	223(38.4%)
Two times in a week	81(13.9%)
Once a week	133(22.9%)
< =Once in 2 weeks	144(24.8%)
**Why have you left home?**	
Only for essentials (such as shopping, going to the doctor, etc.)	514(78.7%)
Even for unnecessary work (such as visiting relatives and friends, etc.)	69(10.6%)
Have not go out	70(10.7%)
**Work condition**	
Left home because of my job	212(32.5%)
working remotely	71(10.9%)
employed but stopped working because of the outbreak	110(16.8%)
Unemployed	260(39.8%)
**To what extent have your co-living complied the quarantine conditions?**	
High	365(55.9%)
Medium	254(38.9%)
Low	26(4%)
Not at all	8(1.2%)
**History of becoming infected with COVID-19**	
Yes	5(0.8%)
No	648(99.2%)
**History of becoming infected with COVID-19 in first-degree family**	
Yes	12(1.8%)
No	641(98.2%)

The fourth section of the questionnaire evaluated the participants’ practice in terms of their role in violent behaviors as a perpetrator or victim. This section includes 12 questions that are intended to focus on being abused by or abusing family members and spouses in five categories of violence, namely emotional, verbal, sexual, and physical. Each question was scored using a two-point Likert scale (0 = no increase; 1 = increase). The questionnaire’s validity was approved by Shiraz University of Medical Science academics and professionals, including psychologists, sociologists, and public health experts. A pilot study of 50 people was also conducted to assess the questionnaire’s reliability, and a Cronbach alpha of 0.70 was established for the entire questionnaire (knowledge [72%], attitude [88%], and practice [80%]) . It should be noted that any questionnaires that were not entirely filled out or submitted were automatically eliminated.

### Statistical analysis

The data were input into SPSS software (version 25), and the data entry accuracy was checked by randomly selecting data from the software and matching it with the corresponding questionnaires. To determine the mean scores of knowledge, the standard deviation (SD) and mean score (M) for knowledge were calculated. The independent t-test and crosstab were then used on all participants to determine the link between public knowledge and practice and gender, married status, and education level. Furthermore, the graph was utilized to compare the frequency and percentages of individuals (victims and perpetrators) by five types of violence. The primary goal of this study was to determine the relationship between all variables and an increase in the rate of violence during quarantine, hence univariate crosstab analysis was used. The variables with a *p*-value less than 0.2 were then selected for inclusion in the multivariate analysis using forward regression logistic. In the final analysis, a p-value of less than 0.05 was considered statistically significant.

### Ethics

The study was approved by the Ethics Committee of Shiraz University of Medical Sciences (IR.SUMS.REC.1399.164). It was carried out in accordance with the Helsinki Declaration of 1995 and its subsequent revisions (General Assembly of the World Medical Association 2014). Electronic informed consent was obtained from each participant at the beginning of the web-based survey. Participants could withdraw from the survey at any moment without providing any justification.

## Results

Although 696 people completed the questionnaires, only 653 (93.8%) were eventually included in the data analysis owing to missing data. In this study, 64.2% (*n* = 419) of the respondents were between the ages of 31 and 50, and 72.6% (*n* = 474) were female. Moreover, 73.8% (*n* = 482) and 73.0% (*n* = 477) of the participants were married and educated for more than 12 years, respectively. The average number of co-living individuals was 3.31 ± 1.39, and 44.7% (*n* = 292) of them believed that their expenses surpassed their monthly incomes. Table 1 tabulates the demographic information and socioeconomic status of the interviewees.

### Knowledge

The independent t-test analysis regarding knowledge showed that the educational level of higher than 12 years had a positive statistical association with the knowledge of violence (*P* < 0.001); however, there was no association between gender and marital status in this regard (*P* > 0.05) (Out of 12). In addition, the mean scores of knowledge based on marital status were 9.87 ± 1.87 and 9.89 ± 2.00 (out of 12) for the single and married respondents, respectively. Additionally, the mean scores of knowledge according to the educational level were 9.12 ± 2.38 and 10.17 ± 1.84 (out of 12) for the participants with ≤12 and > 12 years of age, respectively.

### Attitude

Crosstab analysis in the attitude section showed that being male had a positive statistical correlation with the attitude toward the need for using violence to solve upcoming problems in the family (*P* < =0.05); however, no association was observed between marital status and educational level in this regard (*P* > 0.05). Furthermore, the education level of those older than 12 years had a significant correlation with the belief that displaying violent behaviors is a good way to show feelings (*P* ≤ 0.05). Nevertheless, no association was observed between gender and marital status in this regard (*P* > 0.05).

### Practice

**Fig. 1:**
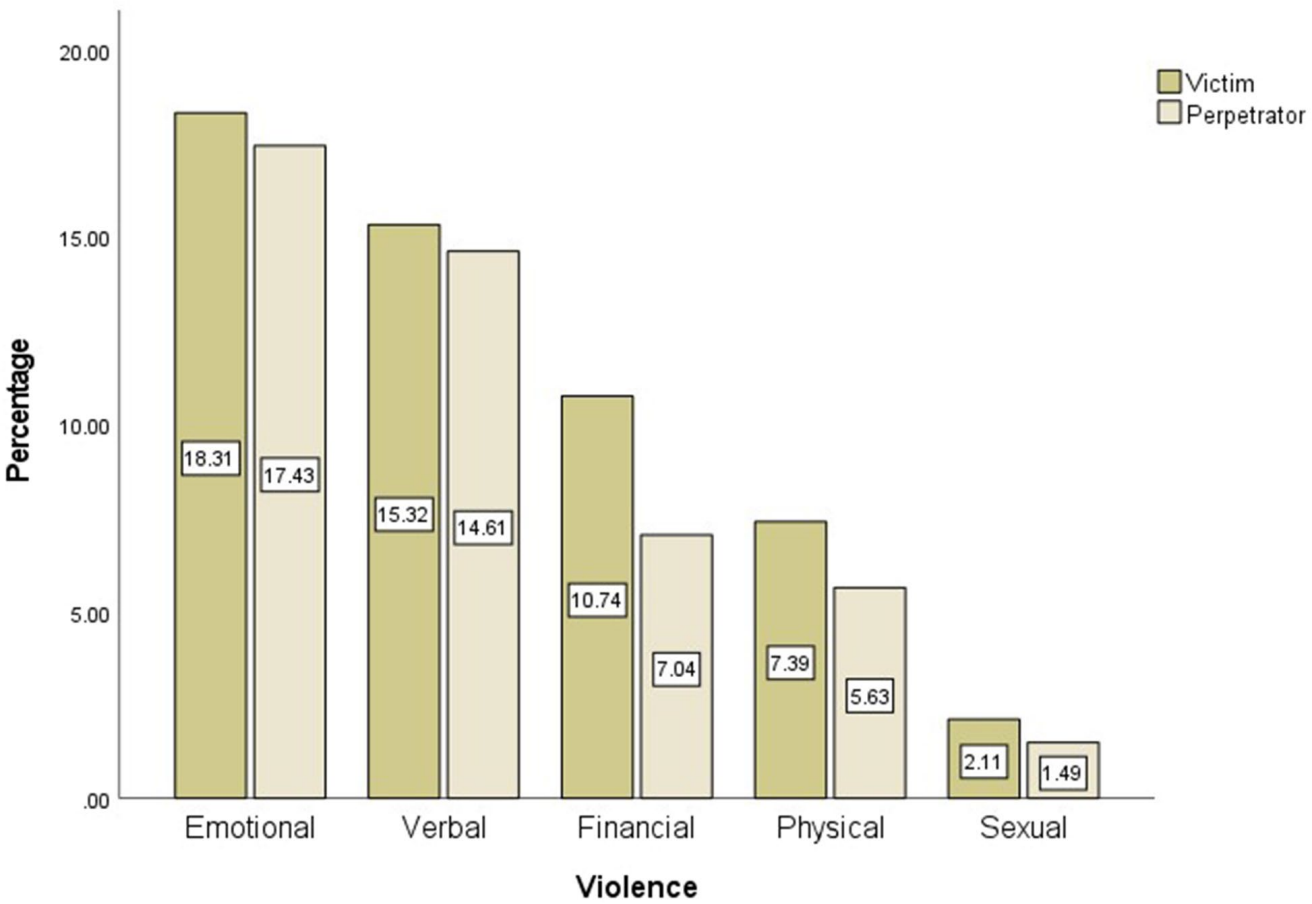
The Percentage of Individuals (Victim & perpetrator) by Five Types of Violence

**Fig. 2:**
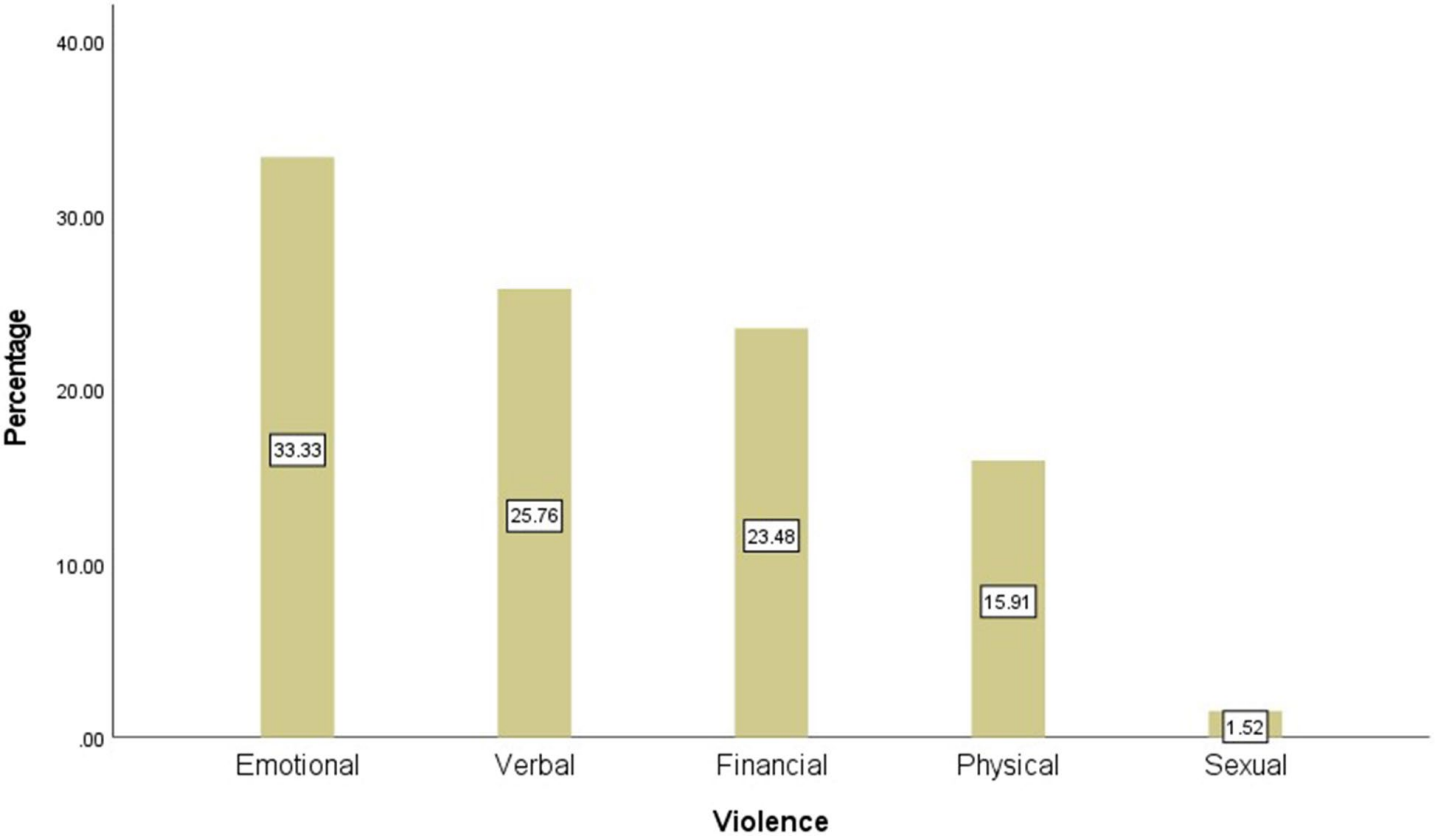
The Percentage of Single Participants by Five Types of Violence

Figure 1 illustrates the percentage of individuals as victims or perpetrators of each type of violence. The emotional type of violence had the highest percentage reported as 18.31 and 17.43% in both victim and perpetrator groups, respectively. However, the sexual type had the lowest frequency reported as 2.11 and 1.41% in both victim and perpetrator groups, respectively. Figure 2 depicts the frequency of DV and its types among the single participants. As illustrated in Fig. 2, the emotional type is the most common (33.33%) type of violence; nonetheless, the sexual type was the least frequent (1.52%) type of DV among the single participants.

**Fig. 3:**
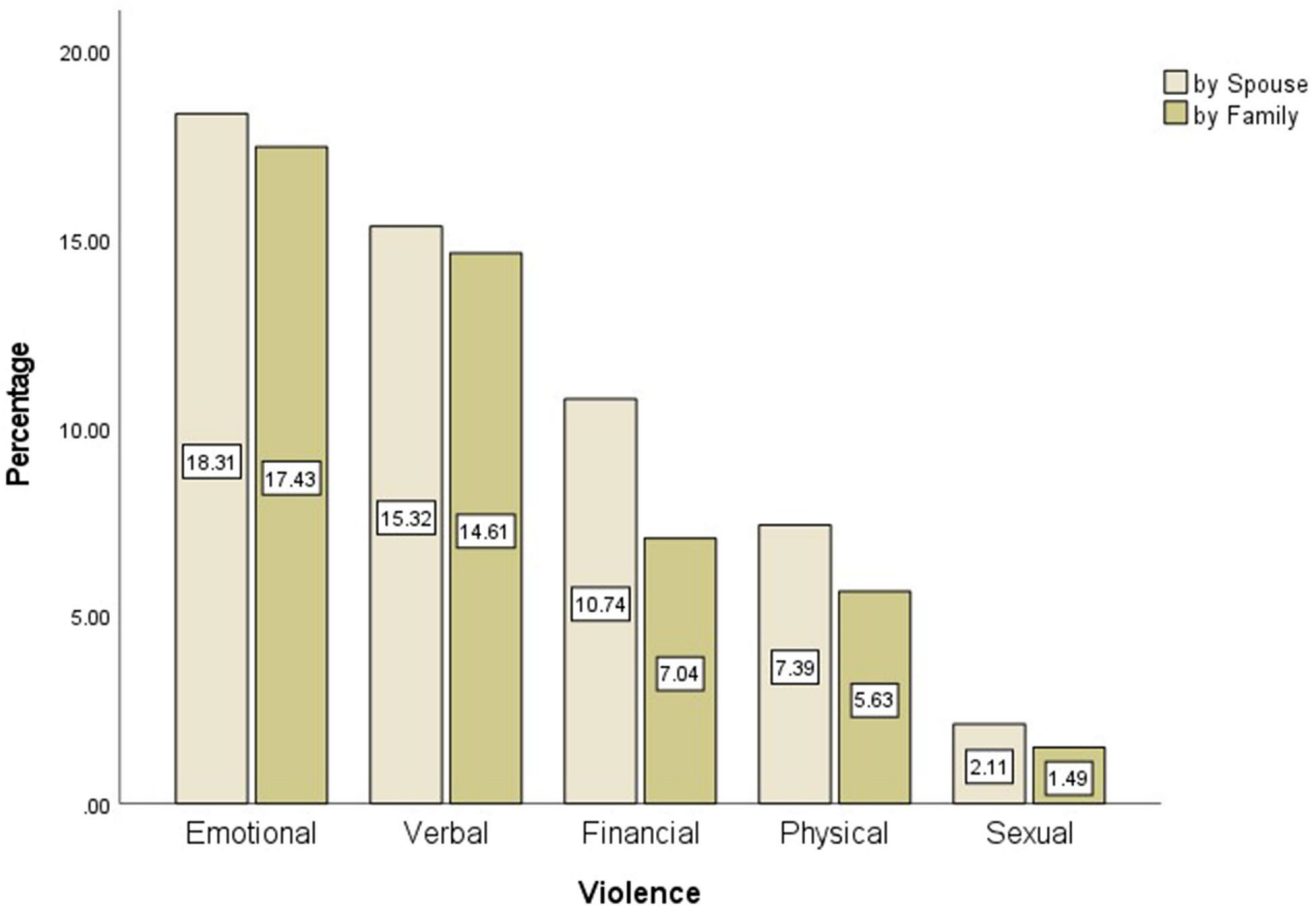
The Percentage of Married Participants by Five Types of Violence Performed by Family and Spouse

Figure 3 depicts the comparison of the frequency of DV in married participants based on the five types of violence committed by their families and spouses. As shown in Fig. 3, the emotional type of DV has the highest prevalence, which was committed by both families (15.16%) and spouses (22.42%). In addition, the sexual type of violence was reported with the least frequency as 3.08 and 0.044% committed by both spouses and families, respectively. Furthermore, most types of DV among the married participants were committed by spouses in comparison to those reported for families.

Univariate analysis showed that 37.5% (*n* = 245) of the participants believed that the rate of violence increased during the quarantine period. Moreover, a positive and significant association was observed between increasing the rate of violence with a first-degree relative involved by COVID-19 (OR = 2.4; *P* = 0.007), remote communication with first-degree relatives more than once a week instead of face-to-face communication (OR = 2.4; *P* = 0.033), infection with COVID-19 (OR = 2.3; *P* = 0.049), lower level of physical activity during the quarantine than before (OR = 2.2; *P* = 0.013), and not going out (OR = 2.1; *P* = 0.016; Table 2). Furthermore, there was a positive and significant association between increasing the rate of violence with a high level of co-living observation of health protocols (OR = 2.0; *P* = 0.007), drug use (OR = 1.8; *P* = 0.033), consumption of alcohol as a beverage (OR = 1.2; *P* = 0.022), and use of tobacco (OR = 1.3; *P* = 0.043; Table 2). Other socioeconomic and demographic information of the subjects was not correlated with the violence (*P* > 0.05; Table 2).

**Table 2:** Univariate Analysis Showing Association between Variables with Violence

Characteristic	Violence		p-value	OR (CI 95%)
	No-Increase n (%)	Increase n (%)		
**How have you changed the state of physical activity?**			0.013	
Higher	56(13.7)	32(13.1)		Ref
No change	145(35.5)	55(22.4)		1.2(1.1–1.8)
Lower	207(50.7)	158(64.5)		2.2(1.4–3.3)
**Have you used tobacco (cigarettes, hookah, and pipe)?**			0.043	
Yes	48(11.8)	44(17.9)		1.3(1.1–1.6)
No	360(88.2)	201(82.0)		Ref
**Have you used alcohol as a beverage?**			0.022	
Yes	8(1.9)	51(20.8)		1.2(1.1–1.8)
No	400(98.1)	194(79.2)		Ref
**Have you used drugs?**			0.033	
Yes	3(0.7)	7(2.9)		1.8(1.1–3.1)
No	405(99.3)	238(97.1)		Ref
**How often have you had remote communication with first-degree relatives who were living in other houses instead of face to face communication?**			0.033	
> 1 time in a week	326(79.9)	174(71.0)		2.4(1.6–3.6)
1 time in a week	47(11.5)	42(17.1)		2.0(1.2–3.3)
Once in a two weeks	14(3.4)	11(4.5)		Ref
< Once in a two weeks	21(5.1)	18(7.3)		1.5(1.2–1.9)
**For what reasons have you left home**			0.016	
Only for essentials (such as shopping, going to the doctor, etc.)	334(81.5)	180(73.5)		1.8(1.1–2.3)
Even for unnecessary work (such as visiting relatives and friends, wandering the streets and sightseeing ...)	37(9.1)	32(13.1)		Ref
Have not go out	37(9.1)	33(13.5)		2.1(1.2–3.5)
**To what extent have your co-living complied**			0.007	
High	245(60.0)	120(49.0)		2.0(1.7–2.3)
Medium	145(35.5	109(44.5)		1.8(1.5–2.0)
Low	14(3.4)	12(4.9)		1.3(1.0–1.8)
Not at all	4(1.0)	4(1.6)		Ref
**Have you got infected corona disease**			0.049	
Yes	1(0.2)	4(1.6)		2.3(1.5–2.9)
No	407(99.8)	241(98.4)		Ref
**Have anyone in your first-degree family got infected corona disease**			0.007	
Yes	3(0.7)	9(3.7)		2.4(1.9–3.2)
No	405(99.3)	236(96.3)		Ref

Multivariate analysis showed that a positive and significant association was observed between the increasing prevalence of DV with infection with COVID-19 (OR = 4.9; *P* = 0.018), drug use (OR = 4.1; *P* = 0.044), high level of the observation of health protocols by family members (OR = 2.5; *P* = 0.004), and lower level of physical activity during the quarantine in comparison to that reported before that (OR = 2.1; *P* ≤ 0.001; Table 3).

**Table 3:** Multivariate Logistic Regression (Forward Wald) Analysis Showing Association of Variables with Violence

Characteristic	Violence		p-value	OR (CI 95%)
	No-Increase n (%)	Increase n (%)		
**Have you got infected corona disease during this period (March 2020 to the end of April 2020)?**			0.018	
Yes	3 (0.7)	9 (3.7)		4.9 (1.3–8.7)
No	405 (99.3)	236(96.3)		Ref
**Have you used drugs?**			0.044	
Yes	3 (0.7)	7 (2.9)		4.1 (1.0–6.8)
No	405 (99.3)	238 (97.1)		Ref
**How have you changed the state of physical activity during the Corona quarantine period (March 2020 to the end of April 2020) compared to before ؟**			< 0.001	
Higher	56 (13.7)	32 (13.1)		Ref
No change	145 (35.5)	55 (22.4)		1.2 (1.1–2.0)
Lower	207 (50.7)	158 (64.5)		2.1 (1.4–3.1)
**To what extent have your co-living complied the quarantine conditions during this period (March 2020 to the end of April 2020)?**			0.004	
High	245 (60.0)	120 (49.0)		2.5 (2.0–3.1)
Medium	145 (35.5)	109 (44.5)		1.8 (1.3–2.2)
Low	14 (3.4)	12 (4.9)		1.4 (1.1–1.8)
Not at all	4 (1.0)	4 (1.6)		Ref

## Discussion

The current study focused on the rate of DV associated with the COVID-19 pandemic in the south of Iran. Iran was the third country affected by this pandemic and has suffered from a high rate of COVID-19 mortality up to now [20]. The results of the present study showed a nearly one-third-increasing rate of DV during the lockdown period. Similarly, using 911 call records, McCrary and Sanga studied the impact of the coronavirus lockdown on domestic violence and found that domestic violence increased by about 12% on average and 20% during working hours. In another study, Piquero et al. provided some evidence for an increase in DV as a short-term spike in 2 weeks after a stay-at-home order in Dallas, Texas [21]. A similar situation is also reported in Bangladesh [22] and Tunisia [23].

Furthermore, among different types of violence, emotional, verbal, and financial violence were the most commonly reported types, respectively. The obtained findings of this study also demonstrated that infection with COVID-19 and drug use in the lockdown period had the strongest associations with the increasing rate of violence, compared to other factors.

Increasing rates of psychological issues have been recorded in several studies during natural and man-made disasters and crises, such as the Ebola pandemic [24]. According to the findings of a study on the psychological impacts of severe acute respiratory syndrome, post-traumatic stress disorder (PTSD) was the most common psychological disorder in the long-term follow-up [25]. The outcomes of the aforementioned studies revealed a rising tendency of psychological issues during pandemics over the world.

A review of previous outbreaks showed that changing individuals’ lifestyles (e.g., through quarantine, lockdown policies, and physical distancing) might have adverse effects, such as panic buying and hoarding, incidents of racism, the psychological pressure of productivity, marginalization, and violence [26].

Approximately, regarding the COVID-19 pandemic and concurrent with the social distancing and home isolation policies, the reports of increasing domestic or intimate violence have been recorded in different countries. In Brazil, a 40–50% increase was reported in the rate of violence [15]. Piquero et al. (2021) in a meta-analysis, by analyzing eighteen empirical studies and 37 estimates, found that in response to lockdown policies in the course of the COVID-19 pandemic, a significant increase between pre-and post- lockdown policies could be seen [21]. These results are aligned with our study, as we reported that about one-third of the increasing rate of DV has been reported by our participants. However, despite our results, a study in Canada has reported a nearly 33% reduction in emergency department admissions for sexual assault and domestic violence rather than pre-COVID-19 [27]. Similarly, in Australia, the results of a study showed that although the reports of crimes have reduced by 40%, the rate of domestic abuse call-outs increase by 5% during the initial months of the COVID-19 pandemic [28]. This increasing trend of violence can also be observed in other countries, such as China, the United Kingdom, the United States, France, Argentina, Cyprus, and Singapore [19, 28–30]. In the United Kingdom, between March 23 and April 12, 2020, women’s mortalities due to the use of violence against them increased more than two fold, up to 16 mortalities, compared to the average rate of the last 10 years [31].

The present study assessed the levels of knowledge of violence and its types and individuals’ attitudes toward DV. Subjects with a higher level of education appear to be more aware of the meaning of violence and its behavioral manifestations, as expected. The participants had more negative attitudes toward using violence as a way to express their feelings than those with less than 12 years of education. It seems that further knowledge of violence may be achieved during the period of academic learning. In addition, further knowledge and higher educational levels help individuals to have a more constructive attitude toward violence and use healthier behaviors to express their feelings.

On the other hand, as previously mentioned, more men than women believed that violence could be committed as a way to solve family problems. The aforementioned issue, if combined with a lack of anger management and problem-solving skills, can facilitate the process of committing violence by men who believe they are allowed to use violence to solve their family problems. In line with the results of previous studies conducted in Iran [32–34] and another study carried out in Rwanda [35], the findings of the present study showed that emotional violence was the most common type of violence. This result is inconsistent with the finding of a study performed by Ahmadi et al. in this regard. They reported that the prevalence of physical and emotional violence was almost similar among Iranian female victims [12].

In another study carried out by Acierno et al. on elderly respondents, it was demonstrated that current financial abuse by a family member was the most common type of violence in adults aged 60 years or older [36] In all the above-mentioned studies [17, 36–38], sexual violence was the least common among different types of DV, which is consistent with the results of the present study.

Previous studies on DV have collected mainly self-reporting data from victims. A study conducted by Zamorski et al. evaluated the role of a perpetrator or victim in different types of violence (i.e., physical, sexual, emotional, and financial) in intimate partner violence among married men [39]. In line with the findings of the aforementioned study [39], the present study showed that the victims reported much more experience of violence than the perpetrators. Moreover, perpetration and victimization of physical and sexual violence were less frequent in emotional and financial violence. Furthermore, in the present study, because the married subjects spent more time with their spouses during the quarantine, they reported more violence (regarding all five types), compared to other family members.

In the current study, it was also observed that the most important variables that were significantly associated with an increase in the rate of violence were drinking alcohol, smoking tobacco, and less going out. It seems that staying together for a long time due to pandemic conditions and home quarantine can cause more tensions in families. A lack of problem-solving skills and use of avoidance strategies, such as drinking alcohol and smoking tobacco, can also lead to an increase in DV [14–16]. One of the main reasons for this relationship is that when individuals adopt further avoidance strategies, more family issues remain unresolved, and finally, individuals may use violence to solve family problems. On the other hand, drinking alcohol may cause mood changes and irritability, which may lead to DV. In line with the results of the current study, the results of other studies showed an association between the consumption of alcohol and an increase in DV [39–43].

Multivariate analysis revealed that four variables, including infection with COVID-19, high level of coliving observation of health protocols, lower level of physical activity, and drug use, had a positive and significant association with increasing the rate of DV. It seems that infection with COVID-19, quarantine, and anxiety and worry about the consequences of the disease may cause a great deal of stress and lead to aggression and violence. In addition, a high level of observing preventive routes in coliving and showing too much sensitivity to follow these protocols can cause disagreement and family conflicts. Moreover, decreased physical activity in the quarantine period may have a negative effect on mental health, increase anxiety and depression, and lead to DV by lowering the tolerance threshold.

In line with the findings of other investigations [40, 42, 43], the results of the present study demonstrated that DV was associated with drug use. Although a small number of participants reported drug use, this variable had a significant effect on the rate of DV. It can be said that drug use, similar to drinking alcohol, is a kind of avoidance strategy, leaving family issues unresolved. On the other hand, drug use may cause numerous mood changes and irritability which may result in DV.

The results of this study suggest an urgent need for government and public support to deal with the physical and psychological effects of domestic violence caused by prolonged lockdown policies.

### Limitations

Because the participants in this study were in quarantine, an online questionnaire was required. Those who did not have access to the internet or cellphones were unable to participate in the study. As a result, the current online survey may not be representative of the overall population. Furthermore, due to several elements on sexual violence in the checklist, the age of 15 was regarded the minimal age for inclusion in the study; as a result, individuals under the aforementioned age were missed. Both the high number of questions and the small smartphone screen posed challenges for participants. However, due to quarantine limitations and the demand that people stay at home, the participation rate was relatively high, and the desired sample size was met in a short period of time.This study was cross-sectional in nature; however, a longitudinal investigation is required to evaluate the many factors influencing violence during the quarantine period.

## Conclusion

In summary, during the COVID-19 pandemic, the rate of DV in Iran increased during the quarantine period. This issue may have both acute and long-term consequences for families and communities. As a result, policymakers should devise a variety of interventional approaches to reduce DV during this pandemic.

## Data Availability

The datasets used and/or analyzed during the study are available on reasonable request from the corresponding author.

## Abbreviation

DV: Domestic Violence
